# Sapovirus Outbreaks in Long-Term Care Facilities, Oregon and Minnesota, USA, 2002–2009

**DOI:** 10.3201/eid1805.111843

**Published:** 2012-05

**Authors:** Lore E. Lee, Elizabeth A. Cebelinski, Candace Fuller, William E. Keene, Kirk Smith, Jan Vinjé, John M. Besser

**Affiliations:** Oregon Public Health Division, Portland, Oregon, USA (L.E. Lee, W.E. Keene);; Minnesota Department of Health, St. Paul, Minnesota, USA (E.A. Cebelinski, C. Fuller, K. Smith);; Centers for Disease Control and Prevention, Atlanta, Georgia, USA (J. Vinjé, J.M. Besser)

**Keywords:** norovirus, sapovirus, Caliciviridae, viruses, gastroenteritis, long-term care, disease outbreaks, Oregon, Minnesota, United States

## Abstract

We tested fecal samples from 93 norovirus-negative gastroenteritis outbreaks; 21 outbreaks were caused by sapovirus. Of these, 71% were caused by sapovirus genogroup IV and 66% occurred in long-term care facilities. Future investigation of gastroenteritis outbreaks should include multi-organism testing.

## Abstract 2

Sapovirus gives new meaning to the phrase “cradle to grave.” Historically, sapovirus has been associated with gastrointestinal illness in children living in group settings such as hospitals, shelters, or refugee camps. But now, sapovirus outbreaks are occurring among elderly residents of long-term care and similar facilities. These elderly residents are especially vulnerable to rapidly transmitted gastrointestinal viruses and serious complications. This virus has been making the rounds in long-term care facilities since 2002, and outbreaks started increasing in 2007. Sapovirus testing should be added to routine diagnostic workups for gastrointestinal infections, regardless of patient age group. Results can be used to develop prevention, control, and treatment guidelines, especially for vulnerable elderly populations.

Viral gastroenteritis outbreaks are associated with illness and death when they occur in institutional settings, notably in long-term care facilities (LTCFs) for the elderly (*1*). Although most reported outbreaks in LTCFs are caused by norovirus (*2*), some have similar epidemiologic characteristics but are norovirus-negative after >2 fecal samples are tested by real-time reverse transcription PCR (RT-PCR). Epidemiologically, these norovirus-like gastroenteritis outbreaks are characterized by 24–48-hour incubation periods, if known; vomiting in >50% of affected persons; and 12–60-hour median illness durations (*3*).

Norovirus and sapovirus are separate genera of the family *Caliciviridae*. Sapovirus was first detected in 1977 as the cause of a gastroenteritis outbreak in a home for infants in Sapporo, Japan (*4*), and was thereafter reported primarily among young children with acute gastroenteritis (*5*). After sapovirus RT-PCR was developed (*6*), sapovirus outbreaks were discovered in LTCFs and other settings populated by adults (*7**–**9*). Sapovirus genogroups I, II, IV, and V (GI, GII, GIV, and GV, respectively) infect humans (*10*). This report describes sapovirus outbreaks in Oregon and Minnesota, USA, during 2002–2009.

## The Study

The Oregon and Minnesota state public health departments investigated 2,161 gastroenteritis outbreaks reported during 2002–2009. Of these, 1,119 (52%) were caused by laboratory-confirmed norovirus (defined as >2 norovirus-positive fecal samples by RT-PCR); 466 (22%) were caused by bacteria, parasites, and other agents; 403 (19%) had no fecal samples to analyze; 142 (7%) were norovirus negative (defined as >2 norovirus-negative fecal samples by RT-PCR) and, when tested, were negative for enteric bacterial pathogens; and 31 (<1%) had a single norovirus-negative stool sample. Outbreak-related fecal samples were archived when any specimen remained after analysis, creating a convenience sample of feces for this and other studies.

The Minnesota Public Health Laboratory tested feces from 93 (66%) of the 142 norovirus-negative outbreaks with RT-PCRs for astrovirus, adenovirus, rotavirus, norovirus, and sapovirus (*6*). Sapoviruses were genotyped by sequence analysis of the capsid gene (*11*).

Defining a sapovirus outbreak in this study as >1 sapovirus-positive fecal sample, 21 (23%) of the 93 norovirus-negative outbreaks were found to be caused by sapovirus. Adenovirus or norovirus were also identified in 4 (19%) of the 21 sapovirus outbreaks (Table 1). The unexpected norovirus finding is likely due to slight variations in testing methods between state public health laboratories and viral loads nearing the detection level of the RT-PCR.

**Table 1:** Microbiology of 21 sapovirus outbreaks, Oregon and Minnesota, USA, 2003–2009*

State	Outbreak no.	Fecal samples, no.		Genotype	Results
		Sapovirus positive	Tested		
MN	2002–438	1	4	IV	Sapovirus only
MN	2002–439	3	5	IV	Sapovirus only
MN	2003–644	1	2	II	Sapovirus only
OR	2004–066	1	2	V	Sapovirus only
MN	2006–924	2	3	IV	Sapovirus only
OR	2007–001	3	8†	IV	Sapovirus, norovirus GI
OR	2007–013	3	3	IV	Sapovirus only
OR	2007–023	3	7	IV	Sapovirus only
OR	2007–028	4	6	IV	Sapovirus only
OR	2007–039	3	4	IV	Sapovirus only
OR	2007–046	4	4	IV	Sapovirus only
OR	2007–086	4	5	IV	Sapovirus only
OR	2007–091	4	6‡	IV	Sapovirus, adenovirus
OR	2007–221	1§	2	I	Sapovirus, norovirus GII
OR	2007–228	1	1	IV	Sapovirus only
OR	2008–109	1	6	I	Sapovirus only
OR	2008–128	3	5¶	I	Sapovirus, adenovirus
MN	2008–1308	3	3	I	Sapovirus only
MN	2008–1327	3	3	IV	Sapovirus only
OR	2009–146	3	3	IV	Sapovirus only
OR	2009–167	2	2	IV	Sapovirus only

*MN, Minnesota; OR, Oregon; G, genogroup. †One norovirus GI–positive sample. ‡One adenovirus-positive sample. §Norovirus GII co-infection. ¶Two adenovirus-positive samples.

Of 21 sapovirus outbreaks, LTCFs accounted for 12 (66%); grade schools for 2 (10%); and a prison, a large psychiatric hospital, a cruise ship, a restaurant, and a bed and breakfast for 5 (24%). During 2007, 10 outbreaks (48%) occurred; 14 outbreaks (67%) occurred during the colder months (November–March) of each observed year. Person-to-person transmission accounted for 18 (86%) of 21 outbreaks. On the basis of the outbreak setting, foodborne transmission was suspected, but not confirmed, in 3 (14%) of 21 sapovirus outbreaks; food items were not implicated. Outbreaks involved 5–44 persons (median 34 persons) per outbreak and lasted 1–28 days (median 15 days) (Table 2).

**Table 2:** Descriptive epidemiology of 21 sapovirus outbreaks, Oregon and Minnesota, USA 2002–2009*

Infection and state	Outbreak no.	Setting	Transmission	Outbreak features		No. cases‡	Symptoms, no. patients		
				Date	No. days†		Vomiting§	Diarrhea§	Fever¶
Sapovirus only									
MN	2002–438	Grade school	Person-to-person	2002 Apr	11	15	NA	NA	NA
MN	2002–439	Long-term care	Person-to-person	2002 Apr	1	34	NA	NA	NA
MN	2003–644	Grade school	Person-to-person	2003 Dec	8	17	NA	NA	NA
OR	2004–066	Long-term care	Person-to-person	2003 Mar	17	44	23	44	8
MN	2006–924	Long-term care	Person-to-person	2006 Feb	13	24	9	24	11
OR	2007–013	Long-term care	Person-to-person	2007 Jan	15	12	7	9	NA
OR	2007–023	Long-term care	Person-to-person	2007 Jan	7	35	16	33	3
OR	2007–028	Long-term care	Person-to-person	2007 Jan	9	12	7	5	6
OR	2007–039	Long-term care	Person-to-person	2007 Jan	22	14	8	12	2
OR	2007–046	Long-term care	Person-to-person	2007 Jan	5	15	11	15	0
OR	2007–086	Long-term care	Person-to-person	2007 Feb	13	8	6	7	NA
OR	2007–228	Long-term care	Person-to-person	2007 Nov	10	34	14	27	NA
OR	2008–109	Long-term care	Person-to-person	2008 Apr	28	24	10	21	NA
MN	2008–1308	Cruise ship	Foodborne suspected	2008 Aug	1	5	3	5	NA
MN	2008–1327	Bed and breakfast	Foodborne suspected	2008 Nov	3	7	2	7	2
OR	2009–146	Psychiatric hospital	Person-to-person	2009 Jul	9	13	9	11	NA
OR	2009–167	Long-term care	Person-to-person	2009 Aug	11	22	7	18	NA
Sapovirus and norovirus									
OR	2007–001	Prison	Person-to-person	2006 Dec	23	154	70	119	1
OR	2007–221	Long-term care	Person-to-person	2007 Nov	16	34	8	29	NA
Sapovirus and adenovirus									
OR	2007–091	Long-term care	Person-to-person	2007 Feb	13	25	15	25	NA
OR	2008–128	Restaurant	Foodborne suspected	2008 Apr	4	26	10	25	NA

*MN, Minnesota; OR, Oregon; NA, data were not collected or could not be analyzed. †Median no. days: 15 (range 1–28 days). ‡Laboratory-confirmed and epilinked cases with systematically collected symptoms; these are not complete case counts. Median no. cases: 34 (range 5–44 cases). §Of 269 patients, vomiting was reported for 132 (49%) and diarrhea for 238 (88%). ¶Of 141 patients, fever was reported for 32 (23%).

Clinical data were available for 141–269 patients from 14 sapovirus outbreaks in which neither adenovirus nor norovirus were identified. Of 141 patients, 32 (23%) had fevers. Of 269 patients, 132 (49%) had vomiting, and 238 (88%) had diarrhea (Table 2). In Oregon, 1 person with sapovirus was hospitalized and 1 died; no hospitalizations or deaths occurred in Minnesota among persons with sapovirus. Symptoms lasted 24–105 hours (median 48 hours) (data not shown).

Four (19%) of 21 sapovirus outbreaks were caused by sapovirus GI, 1 (5%) by sapovirus GII, 15 (71%) by sapovirus GIV, and 1 (5%) by sapovirus GV (Table 1). The genogroup-specific differences between outbreak settings and between the proportions of vomiting, diarrhea, and fever were not statistically significant. Seventy-three percent of sapovirus GIV outbreaks occurred in 2007. A representative sequence from each outbreak was placed in the phylogenic tree (Figure). Of 14 sapovirus outbreaks with >2 sapovirus-positive samples, sequences from 12 were identical within the outbreaks, and 2 had 2 different sequences (Figure).

**Figure:**
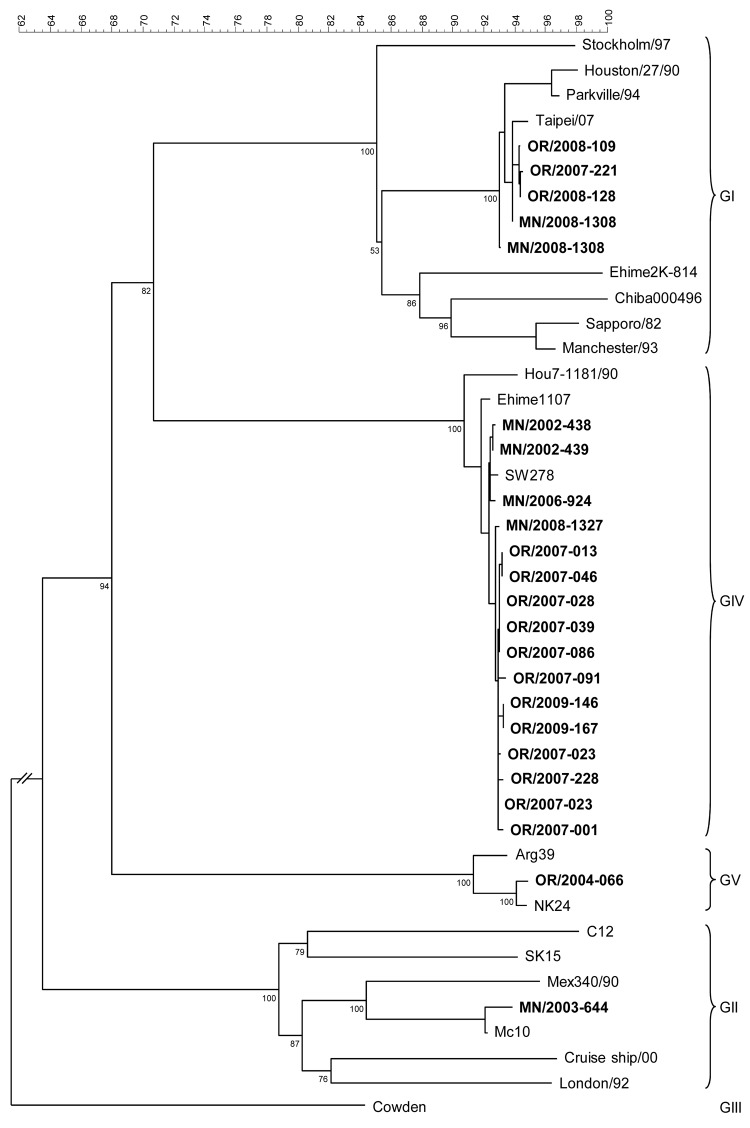
Phylogenetic tree of sapovirus sequences from outbreaks of acute gastroenteritis reported to state public health departments in Oregon and Minnesota, 2002–2009, on the basis of partial capsid nucleotide sequences. Reference strains [GenBank accession numbers] include Sapporo/1982/JP [U65427], Parkville/1994/US[U73124], Stockholm318/1997/SE [AF194182], Chiba000496/2000/JP [AJ606693], Ehime2K-814/2000/JP [AJ606698], London/1992/U K[U95645], Mex340/1990/MX [AF435812], cruise ship/2000/US [AY289804], PEC-Cowden/1980/US [AF182760], Hou7-1181/1990/US [AF435814], and Argentina39/AR [AY289803]. **Boldface** indicates state-assigned outbreak identification numbers. Scale bar represents percent genetic similarity between sequence types. Genogroups are indicated on the right. For genogrouping, GenBank sequences of well-characterized genogroups were aligned with outbreak sequences, and a phylogenetic tree was created by the neighbor-joining method by using BioNumerics (Applied Maths, Austin, TX, USA). Genotypes were assigned on the basis of >95% similarity to reference strains. Outbreak strain sequences were deposited in GenBank under accession nos. HM800902–HM800920.

## Conclusions

In this study, the high (66%) proportion of sapovirus outbreaks in LTCFs among 21 outbreaks of previously unknown etiologies is likely to be an artifact of legally mandated outbreak reporting by health care facilities rather than the true distribution of sapovirus outbreaks in Oregon and Minnesota. Still, elderly residents of LTCFs are especially vulnerable to rapid transmission of viral enteric pathogens and serious complications from infection with these agents (*12*), and therefore merit the attention of public health.

Our data, together with a recent study in Canada (*7*), demonstrate that sapovirus has been circulating among the institutionalized elderly since at least 2002 and that sapovirus outbreaks increased in 2007 as part of a worldwide surge in gastroenteritis outbreaks (*2**,**7**,**9*). Before these retrospective studies, sapovirus infections among adults >65 years old had been reported as single cases at a low (3%) rate in 2002 (*13*) and as nosocomial outbreaks in 2010 and 2005 (*8*,*14*). In 2010, Svraka et al. reported an age distribution shift from younger to older persons (*9*).

Sapovirus outbreaks occurred in the same settings and had the same seasonal distribution as norovirus outbreaks (*2**,**15*). Our study adds clinical details to information provided by studies in Canada and Europe (*7**,**9*). The clinical profile of sapovirus outbreaks in this study (49% vomiting, 88% diarrhea, and 23% fever, plus a median duration of 48 hours) approximates the criteria of Kaplan et al. (*3*), which are still used to evaluate norovirus outbreaks when laboratory resources are limited. We found, however, that sapovirus and norovirus outbreaks are clinically and epidemiologically similar enough to be indistinguishable without laboratory testing.

This study has at least 3 limitations. First, testing a convenience sample of fecal specimens from norovirus-negative outbreaks might have introduced selection bias, the impact of which is uncertain. Second, because outbreak reporting from institutions other than LTCFs is not legally mandated, outbreaks in these settings are underreported. Third, feces from norovirus-positive outbreaks were not assayed for sapovirus. Previously undetected norovirus GI and GII discovered among 21 sapovirus outbreaks indicates that outbreaks might have had >1 etiology. It is therefore likely that the number of sapovirus outbreaks was underestimated.

In summary, gastroenteritis outbreaks in LTCFs should be investigated by public health departments in conjunction with testing of fecal specimens. Public health laboratories should archive fecal samples from all gastroenteritis outbreaks until a cause is established. As in this study, testing with assays for sapovirus, astrovirus, adenovirus, and rotavirus should be conducted when standard methods for norovirus and enteric bacterial pathogens fail to identify a causative agent.

In keeping with recent recommendations, at minimum, adding sapovirus to routine diagnostics of infections that occur in any setting and by any mode of transmission will establish etiologies of some norovirus-negative outbreaks and help define the disease impact and clinical characteristics of sapovirus infections (*9**,**10**,**13*). These data can in turn be used to develop and evaluate sapovirus disease management guidelines and sapovirus outbreak prevention and control measures.
